# Is there any association between contrast-induced nephropathy and serum uric acid levels?

**DOI:** 10.34172/jcvtr.2021.20

**Published:** 2021-02-20

**Authors:** Fardin Mirbolouk, Samira Arami, Mahboobe Gholipour, Yasaman Khalili, Seyedeh Shiva Modallalkar, Mona Naghshbandi

**Affiliations:** ^1^Cardiovascular Diseases Research Center, Department of Cardiology, Heshmat Hospital, School of Medicine, Guilan University of Medical Sciences, Rasht, Iran; ^2^Rajaei Cardiovascular Medical and Research Center, Iran University of Medical Sciences, Tehran, Iran

**Keywords:** Uric Acid, Hyperuricemia, Acute Kidney Injury, Contrast Nephropathy

## Abstract

***Introduction:*** During the recent years, several studies have investigated that hyperuricemia is associated with greater incidence of contrast induced nephropathy (CIN). Most of them are in acute conditions like primary percutaneous coronary interventions. This study aimed to assess the relationship between high serum uric acid and incidence of acute kidney injury in patients undergoing elective angiography and angioplasty.

***Methods:*** This prospective study was conducted on 211 patients who were admitted to hospital for elective coronary angiography or angioplasty. The researchers measured serum creatinine and uric acid on admission and repeated creatinine measurement in 48 hours and seven days after the procedure. According to serum uric acid, the patients were divided into two groups; group 1 with normal uric acid and group 2 with hyperuricemia which was defined as uric acid more than 6 mg/dL in women and 7 mg/dL in men. CIN is defined as an increased creatinine level of more than 0.5 mg/dL or 25% from the baseline in 48 hours after the intervention.

***Results:*** In total, 211 patients with mean age of 60.58 years were enrolled in the study. Of these, 87 (41.2%) patients were in the high uric acid group and 124 (58.8%) were in the normal uric acid group. CIN was occurred in 16 patients (7.5%). Seven out of 16 (8.04%) were in the high uric acid and nine (7.2%) were in the normal uric acid group. There were no significant differences between the two groups (*P* =0.831).

***Conclusion:*** The frequency of CIN development was not different in the patients with hyperuricemia.

## Introduction

Contrast induced nephropathy (CIN) is one of the most common problems of cardiac catheterization that associated with short and long term mortality and morbidity.^1,2^

Renal failure requiring hemodialysis after coronary intervention is associated with 40% of in-hospital mortality and 2-year mortality rate was 80%.^3^ Most clinical studies described contrast induced nephropathy as an elevation in the creatinine serum level more than 0.5 mg/dL or 25% increase over the baseline during 48-72 days after the procedure.^4-7^ CIN prevention is essential because of no proven effective treatment.^8^ Therefore, the screening of high risk patients based on their risk factors helps clinicians to have opportunities to prevent AKI and improve outcomes.^9-11^

The major complications occur in up to 25% of patients undergoing coronary angiography and angioplasty depending on the presence of the known risk factors including diabetes mellitus, old age, heart failure, anemia, hypertension, hypotension, hypovolemia, low left ventricular ejection fraction (LVEF), acute situation, high contrast volume, contrast osmolality, ionization and as the most important one chronic kidney diseases. ^12-14^

Enough hydration with isotonic crystalloid solutions in the high risk patients is used as the most effective prevention method before and after the procedure.^15,16^

During the recent years, several studies have investigated that hyperuricemia is associated with the greater rate of CIN occurrence.^17-19^ Uric acid is the final product of purine metabolism ^20-22^ and when it rise in the serum is associated with endothelial dysfunction, inflammation, activation of RAAS, inhibition of nitric oxide (NO) system and increased oxidative stress.^23,24^ A new review article suggested that uric acid is a novel independent predictor of CIN and measurement of uric acid level before angioplasty may be a useful method for assessing the risk of developing CIN and short term outcomes. ^19^ However, many studies have been performed in this regard but conflicting results have been reported and uric acid is not a routine attractive test before angiography in our center. Most of the articles in this subject are about acute condition like ST elevation MI (STEMI) or acute coronary syndrome.^5,7,19,25-27^ The important point of this study compared to other studies was as which this study examined just elective procedures and excluded all the primary PCI and urgent angiography cases. Meanwhile, the present study had enough time to eliminate the nephrotoxic drugs and give enough prophylaxis hydration to all the patients. In addition, the researchers failed to enter patients with stage 4 & 5 CKD. Overall, this study had lower risk patients with proper preparation for the procedure.

## Materials and Methods

This prospective study was conducted on 211 patients who were admitted to hospital for elective coronary angiography or angioplasty during 2018-2019 at Dr. Heshmat Treatment and Educational Heart Center in Rasht city.

Exclusion criteria were patients with history of end stage renal disease (ESRD), nonsteroidal anti-inflammatory drug (NSAID) usage within the past seven days, metformin usage during the last 24 hours before the procedure, acute gout, malignancy, acute infection, acute decompensated heart failure and the end the patients who require urgent intervention were excluded from the study.

This study measured serum creatinine and uric acid on admission and repeated creatinine measurement in 48 hours and seven days after the procedure. According to serum uric acid, the patients were divided into two groups; group 1 with normal uric acid and group 2 with hyperuricemia. Hyperuricemia was defined as uric acid more than 6 mg/dL in women and 7 mg/dL in men. Coronary angioplasty was detected based on coronary anatomy and the physician preference. In the patients under the intervention, 500 mL normal saline (0.9%) was infused before the procedure and continued at a rate of 1 mg/kg/h in the patients with normal left ventricle ejection fraction (LVEF) and also 0.5 mg/kg/h in those with LVEF less than 40% for the next 12 hours. Iodoxanole (vesipaque) was used during the intervention. CIN is defined as an elevated serum creatinine levels of 25% or more than 0.5 mg/dL in 48 hours compared to baseline. This study repeated creatinine measurement seven days after the procedure to find its prolonged changes and to investigate if the creatinine returns to baseline or remains high in the CIN group.

The present study recorded the patients’ demographic data and their risk factors including age, sex, diabetes, hypertension, dyslipidemia, anemia, the number of stenosed vessels, left ventricle ejection fraction (LVEF), history of myocardial infarction (MI) or coronary arteries bypass graft (CABG) and the amount of contrast used during the procedure.

### Statistical analysis

Data were analyzed in SPSS software (version 21.0; International Business Machines Corp. Armonk, New York, USA). Continues variables were compared using independent sample, *t* test, or Mann-Whitney test depending on the normality of distributions. The *P* value for the categorical variables was calculated with the chi-square test. A two-tip tailed *P* value of < 0.05 was considered as significant.

## Results

A total of 211 patients were included in the study with mean age of 60.58±11.6 years. All of the patients were admitted for elective coronary angiography. Coronary angioplasty was detected based on coronary anatomy and the physician preference in 62 (29.4%) patients. Of these, 155(73.5%) the patients had good kidney function with GFR>60 mL/min, 87 (41.2%) patients were in the high uric acid and 124 (58.8%) in the normal uric acid group (median (IQR) uric acid= 7.5 (7-8.4) vs. 5 (4.1-5.7), *P* value < 0.001) (Table 1).

**Table 1:** The demographic and clinical data of the patients based on uric acid

	**Total**	**Normal uric acid**	**High uric acid**	***P*** ** value**
Male	134 (63.5%)	80(62%)	54(64%)	0.71
Diabetes	69 (32.7%)	40(32%)	29(33%)	0.87
Hypertension	134 (63.55%)	75(67%)	59(60%)	0.27
Smoking	61 (28.9%)	39(31%)	22(25%)	0.33
History of infarction	5 (2.4%)	2(1%)	3(3%)	0.39
LVEF < 40%	41(19.4%)	19(29%)	22(44%)	0.08
CABG	7 (3.3%)	4(3%)	3(3%)	1
Angioplasty	62(29.4%)	37(29.8%)	25(28.7%)	0.72
Three vessel disease	76 (36.0%)	42(33%)	34(40%)	0.36
Hyperlipidemia	88(42%)	47(38%)	41(47%)	0.18
Anemia	102(48%)	52(41%)	50(57%)	0.026
Age, y	60.58±11.6	59.00±11	61.00±11	0.185
SBP, mm Hg	126.87±19.88	128.00±21	123.00±17	0.068
LVEF (%)	44±11	46.00±9	41.00±12	0.022
Blood sugar, mg/dL	156.88± 84.84	162.00±88	149.00±79	0.001
Baseline Cr, mg/dL	Median 1.04 IQR(92-117)	Median 0.98 IQR (0.9-1.09)	Median 1.1 IQR(1.01-1.25)	<0.001
In 48 hours Cr, mg/dL	Median 1.06 IQR (93-1.2)	Median 1 IQR(0.92-1.1)	Median 1.16 IQR(1-1.37)	<0.001
In 7 days Cr, mg/dL	Median 1.04 IQR(0.92-1.2)	Median 1 IQR(0.9-1.1)	Median 1.1 IQR(1-1.29)	<0.003
Uric.acid, mg/dL	Median 6 IQR(6-7.3)	Median 5 IQR(4.1-5.7)	Median 7.5 IQR(7-8.4)	<0.001
Baseline GFR, mL/min	76.96±24.96	23.74±81.46	24.18±70±56	<0.001
In 48 hours GFR, mL/min	76.75±24.54	23.75± 81.09	24.45±70.56	<0.002
In 7 days GFR, mL/min	76.01±24.97	24.35±79.98	24.88±70.34	<0.006
Contrast volume, mL	Median 150 IQR (150-200)	Median 150 IQR (150-200)	157.00, IQR(150-200)	0.098

Abbreviations: CABG, coronary arteries bypass graft; Cr, creatinine; GFR, glomerular filtration rate; IQR, interquartile ranges; LVEF, left ventricle ejection fraction; SBP, systolic blood pressure; The values are expressed as the mean ± SD or the median with the interquartile ranges or the numbers with percentages. *P* value < 0.05 was considered significant.

The demographic and clinical data of the patients and the two groups are presented in Table 1. The frequency of men (64% vs 62%, *P* value = 0.71), diabetes(33% vs. 32%, *P* value = 0.87), history of MI(3% vs 1%, P value = 0.39), history of CABG ( 3% vs. 3%, *P* value =1), heart failure with reduced ejection fraction(44% vs. 29%, *P* value = 0.08), three vessel disease(40% vs 33%, *P* value = 0.36), hyperlipidemia (47% vs. 38%, *P* value = 0.18), hypertension(59% vs. 75%, *P* value = 0.27), smoking(25% vs. 31%, *P* value = 0.33), angioplasty (29.8% vs. 28.7%, *P* value = 0.72), volume of contrast used in the procedures with interquartile range (IQR)=(157 (150-200) vs. 150(150-200) , *P* value = 0.098), mean age ( 61±11 vs. 59±11, *P* value = 0.185) and systolic blood pressure at the time of procedure (123±17 vs. 128±21, *P* value = 0.27) had no significant differences between the groups.

The hyperuremic patients had lower blood sugar at the time of procedure (149±79 vs. 162±88, *P* value < 0.001) but the anemia was more prevalent in this group (57% vs.41%, *P* value = 0.026) and they had lower LVEF (41±12 vs. 46±9, *P* value = 0.022).

The hyperurecemic group had higher baseline creatinine (Median (IQR)= 1.1(1.01-1.25) vs. 0.98(0.9-1.09), *P* value < 0.001) and lower baseline glomerular filtration rate (GFR)(70.56± 24.18 vs. 81.46±23.75, *P* value < 0.001). (Table 1)

Serum creatinine and GFR were compared in 48 hours and seven days after the procedure and this study found that the patients with high uric acid had still higher creatinine and lower GFR at both times, so that median (IQR) of creatinine was 1.16 (1-1.37 vs. 1(0.9-1.1), *P* value < 0.001 in 48 hours and 1.1(1-1.29 vs 1(0.9-1.1), *P* value < 0.003 in 7 days and mean±SD of GFR was 70.56±24.45 vs. 81.09±23.75, *P* value < 0.002 in 48 hours and 70.34±24.88 vs. 79.98±24.35, *P* value < 0.006 in seven days. (Table 1)

**Figure 1:**
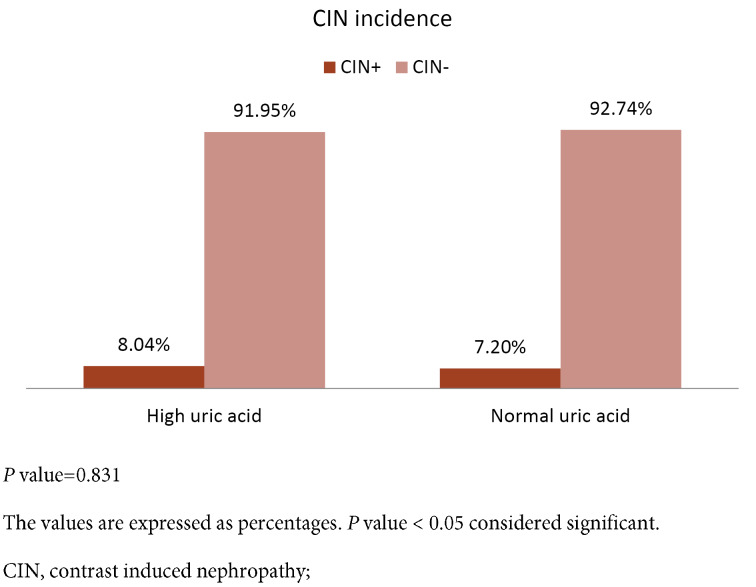
Comparison of CIN occurrence between patients with high and normal serum uric acid

CIN occurred in 16 patients (7.5%); seven patients of whom had (8.04%) high uric acid and nine (7.2%) had normal uric acid. There were no significant difference between them (*P* value = 0.831). (Figure 1) Since most of the studies were in PCI patients, this study repeated the analysis in this subgroup,= again; among PCI group, 37 patients had normal uric acid, 2 (5.4%) cases developed CIN and 25 patients had high uric acid, while three patients (12%) developed CIN. (*P* value = 0.350) Thus, the occurrence of CIN in our PCI patients was not significantly different in uric acid groups.

Furthermore, this study compared other known risk factors of the kidney injury between the patients who developed CIN and the others. The frequencies of men (68% vs. 63%, *P* value = 0.65), age ≥65 years (56% vs 34%, *P* value = 0.231), hypertension (64% vs. 63%, *P* value = 0.93), diabetes (32% vs. 31%, *P* value = 0.89), smoking (37% vs. 28%, *P* value = 0.43), three vessel disease (50% vs. 35%, *P* value = 0.24), left ventricle ejection fraction less than 40% (44% vs. 35%, *P* value = 0.58), coronary bypass graft (CABG) (6% vs. 3.1%, *P*value = 0.5), angioplasty (29.2% vs. 31.2%, *P* value = 0.882) anemia (50% vs. 48%, *P* value = 0.65) and the volume of the used contrast with median (IQR)=150 (150-200) vs. 150(150-200), *P* value = 0.607) had no significant differences in CIN groups. (Table 2).

**Table 2:** Comparison of the known risk factors of the acute kidney injury between patients who developed CIN and those without CIN occurrence.

**Variables**	**Total**	**CIN -**	**CIN +**	***P*** ** value**
Male	134 (63.5%)	123(63%)	11(68%)	0.65
Age ≥65	80(38%)	72(34%)	9(56%)	0.231
Diabetes	69 (32.7%)	64(31%)	5(32%)	0.89
Hypertension	134 (63.55%)	124(63%)	10(64%)	0.93
Smoking	61 (28.9%)	55(28%)	6(37%)	0.43
Anemia	102(48%)	94(48%)	8(50%)	0.65
LVEF < 40%	41	37(35%)	4(44%)	0.58
CABG	7 (3.3%)	6(3.1%)	1(6%)	0.5
Angioplasty	62 (29.4%)	57(29.2%)	5(31.2%)	0.885
Three vessel disese	76 (36.0%)	68(35%)	8(50%)	0.24
Contrast volume	Median150 IQR(150-200)	Median150 IQR(150-200)	Median150 IQR(150-200)	0.607

Abbreviations: CABG, coronary arteries bypass graft; CIN, contrast induced nephropathy; IQR, interquartile range; LVEF, left ventricle ejection fraction The values are expressed as the numbers and percentages, the mean ± SD or the median and the interquartile ranges. *P* value < 0.05 considered significant

Baseline creatinine (0.97 (0.83-1.09) vs. 1.04 (0.92-1.17), *P*value = 0.086) and GFR (69.76±29.69 vs. 78.25±24.01, *P* value = 0.766 ) was not significantly different in the CIN groups but in 48 hours post procedure patients who developed CIN had significantly higher creatinine (1.31(1.15-1.59) vs. 1.05(0.93-1.18), *P* value < 0.001) and lower GFR (60.98±34.02 vs. 76.68±23.71, *P* value < 0.001) and these differences remained even after 7 days between creatinine (1.15(1-1.66) vs. 1.03(0.91-1.18), *P* value = 0.02) and GFR (61±26 vs. 77±24, *P* value = 0.034). (Table 3)

**Table 3:** Changes of creatinine and GFR during 48 hours and 7 days post procedure in the CIN groups

**Variables**	**Total**	**CIN -**	**CIN +**	***P*** ** value**
Creatinine before procedure, mg/dL	Median 1.04 IQR(0.92-1.17)	Median 1.04 IQR(0.92-1.17)	Median 0.97 IQR(0.83 – 0.9)	0.086
Creatinine 48 hours post procedure, mg/dL	Median 1.06 IQR (0.93-1.2)	Median 1.05 IQR (0.93-1.18)	Median 1.31 IQR(1.15-1.59)	<0.001
Creatinine 7 days post procedure, mg/dL	Median 1.04 IQR(0.92-1.18)	Median 1.03 IQR(0.91-1.18)	Median 1.15 IQR(1-1.66)	0.02
GFR before procedure, mL/min	76.96±24.96	24.01±78.25	29.69± 69.86	0.766
GFR 48 hours post procedure, mL/min	76.75±24.54	23.71± 76.68	60.98±34.02	0<001
GFR 7 days post procedure, mL/min	76.01±24	77±24	61±26	0.034

Abbreviations: GFR , glomerular filtration rate; IQR, interquartile ranges

## Discussion

In the present study, CIN occurred in 7.5% of the patients with no significant difference in the high uric acid group. This finding is not consistent with most of the previous studies being reviewed. A recent meta-analysis reviewed 10 articles with 6705 patients and found CIN occurred in 11.5% of the patients after PCI. In this study, baseline serum uric acid level was significantly higher in those who developed CIN. High creatinine level, old age, diabetes mellitus and hypertension were the other significant risk factors.^19^ Likewise, a study by Abdollah et al conducted on 146 patients with acute STEMI underwent primary PCI concluded that 8.8% of the patients experienced developed CIN, 14.1% hyperuricemia and 2.94% with normal uric acid had a significant difference and concluded that elevated uric acid was associated with higher risk of CIN in the STEMI patients with normal serum creatinine after PCI.^25^

This conflict can be explained so that that the patients in the present study had generally lower risk. Most of the patients in the present study had good kidney function as 73.5% had GFR>60 mL/min. This study had no emergent PCI and excluded the patients with acute myocardial infarction. The interventions were elective and the researchers performed enough hydration as prophylaxis to all of the cases before angiography. Most of the procedures were just diagnostic coronary angiography and PCI was performed in only 29% of the cases. Since most of the studies were performed on the PCI patients, the researchers of this study repeated the analysis in this subgroup, again; the occurrence of CIN was not significantly different in uric acid groups. However, maybe if this study had more number of PCI, the results could be different but in the present study, the contrast volume and the number of PCI was the same in the uric acid and CIN groups and did not have an important effect on the results.

The population of this study was somewhat the same as the study by Li et al so that 788 patients with normal creatinine underwent elective PCI with hydration before and after the procedure but in their study CIN occurred with more incidence in the hyperuricemia group (8.1% vs. 1.4%). They demonstrated in hospital mortality and need for renal replacement therapy were significantly higher in hyperuricemia group. Further, other risk factors were older than 75 years , emergent PCI, diuretic usage and need for IABP.^28^

Pakfetrat et al found serum uric acid level failed to differ significantly between CIN+ and CIN- groups. They failed to find a difference between their study groups because their study included a significant number of patients with normal kidney function.^29^ This is in more agreement with this study with most of the patients in favorable kidney function category.

Some of the articles evaluated the effects of hyperuricemia on CIN, especially in CKD patients. Toprak et al conducted an observational study in patients with chronic kidney disease and resulted high serum uric acid can predict CIN.^30^ In another study by Madero et al they observed that hyperuricemia in the patients with stage 3-4 CKD has been appeared to be an independent risk factor of all cause and cardiovascular mortality but not for kidney failure.^31^

Okino et al explored 132 patients with CKD who underwent elective PCI and found that CIN occurred in 6.5% of the patients and incidence of CIN was not significantly different between two uric acid groups, however, they resulted uric acid was a predictor of slow and mild developing factor to renal insufficiency which defined as creatinine rise more than 0.2 mg/dL in 2 weeks after PCI in the patients with CKD.^6^ Likewise, this study repeated creatinine and GFR measurement after seven days and discovered that the patients with CIN had still persistent higher creatinine and lower GFR even after a week post procedure. It indicated the long-term effect of contrast on kidney function. The results were the same in hyperuricemia such that this group had significantly higher creatinine and lower GFR after 7 days. However, this study could not suggest that high uric acid can have a role in this finding. In fact, the patients with hyperuricemia had higher baseline creatinine and lower GFR and these differences were still visible after 48 hours and seven days post procedure. This finding in accordance with the study by Kodama et al found normal uric acid was associated with less renal dysfunction among the adults older than 40 years old and hyperuricemia was associated with diabetic nephropathy.^32,33^ Altogether, it can suggested that high uric acid can reduces renal function independent of our intervention, however, the rise of creatinine post procedure was not enough for leading to CIN.

This study compared other risk factors between the patients with high and normal uric acid. None of the variables like sex, age, diabetes, hypertension, smoking, previous infarction, CABG, coronary anatomy, hyperlipidemia were different between the two groups. Although, the patients had generally lower LVEF in the high uric acid group. Meanwhile, heart failure with reduced ejection fraction had the same frequency in both groups and the frequency of anemia was significantly higher in hyperuricemia patients. The study by Sreenivasan et al exhibited that the severity of anemia was a strong predictor of CIN following coronary angiography^34^ and Li et al also said baseline GFR and anemia were independent risk factors of CIN.^28^ Another study by Eun et al represented that anemia in CKD patients was associated with a 2-fold increase in the risk of hyperuricemia which remained significant even after adjustment for renal function.^35^ The findings of this study are in line with this study about more prevalence of anemia in hyperuricemia patients and they had also less GFR. However, this study had no enough power to suggest this matter while maybe anemia is one of the factors leading to increase the risk of hyperuricemia which can affect kidney function and CIN.

This study failed to evaluate the effects of other risk factors of CIN. The frequency of men, old age, hypertension, diabetes, smoking, baseline GFR, three vessel disease, left ventricle ejection fraction less than 40%, history of CABG and anemia were not meaningfully different in the CIN groups.

The pathophysiology of CIN is complex and multifactorial.^36^ A critical illness can damage the kidney and contrast agent can cause direct tubular toxicity and make kidney injury by renal vasoconstriction, impaired vasodilation, medullary hypoxia and oxidative stress.^37^ Uric acid is the end product of purine metabolism and excrete via kidneys. Uric acid inactivates nitric oxide, an endothelial derived relaxing factor. Therefore, elevated uric acid can induce endothelial dysfunction and renal vasoconstriction.^38^ Further, hyperuricemia accelerates proinflammatory pathways and proliferation of vascular smooth muscle cells and endothelial dysfunction. ^39,40^ Acute kidney injury can occur in acute urate nephropathy with crystal dependent pathways.^36^

In addition, there are several reasons for making uric acid as a significant risk factor of kidney injury and CIN but this study could not show this effect. Furthermore, this study had some limitations.It was a single center study with small samples of patients and short observational period. The researchers performed the study in lower risk patients and procedures, thus so generalization of the results is difficult. Therefore, this study still needs bigger multi center studies to evaluate the effect of hyperuricemia and its treatment on CIN development.

## Conclusion

The frequency of CIN development had no difference in the patients with normal and high uric acid maybe because of lower risk patients and procedures with enough preventive modalities. However, hyperuricemia was accompanied with worse kidney function before and after the procedure.

## Acknowledgements

Hereby, the authors would like to appreciate the vice-chancellor for research and technology of the university, the head and staff members of Dr. Heshmat hospital and all patients who voluntarily participated in this study.

## Competing interest

The authors declare that there are no conflicts of interest regarding the publication of this article.

## Ethical approval

This study is based on the research proposal, approved by the research and technology directorate of Guilan University of Medical Sciences with Ethics Committee code of IR.GUMS.REC.1396.341.

## Funding

This study was financially supported by the research and technology directorate of Guilan University of Medical Sciences , Rasht, Iran (Grant No. 96080610).

## References

[1] Marenzi G, Lauri G, Assanelli E, Campodonico J, De Metrio M, Marana I (2004). Contrast-induced nephropathy in patients undergoing primary angioplasty for acute myocardial infarction. J Am Coll Cardiol.

[2] Rihal CS, Textor SC, Grill DE, Berger PB, Ting HH, Best PJ (2002). Incidence and prognostic importance of acute renal failure after percutaneous coronary intervention. Circulation.

[3] McCullough PA, Soman SS (2005). Contrast-induced nephropathy. Crit Care Clin.

[4] Karabulut A, Sahin I, Ilker Avci I, Okuyan E, Dogan Z, Uzunlar B (2014). Impact of serum alkaline phosphatase level on the pathophysiologic mechanism of contrast-induced nephropathy. Kardiol Pol.

[5] Ma G, Yu D, Cai Z, Ni C, Xu R, Lan B (2010). Contrast-induced nephropathy in postmenopausal women undergoing percutaneous coronary intervention for acute myocardial infarction. Tohoku J Exp Med.

[6] Okino S, Fukuzawa S, Inagaki M, Sugioka J, Ikeda A, Maekawa J (2010). Hyperuricemia as a risk factor for progressive renal insufficiency after coronary intervention in patients with chronic kidney disease. Cardiovasc Interv Ther.

[7] Saritemur M, Turkeli M, Kalkan K, Tanboga İ H, Aksakal E (2014). Relation of uric acid and contrast-induced nephropathy in patients undergoing primary percutaneous coronary intervention in the ED. Am J Emerg Med.

[8] Guo W, Liu Y, Chen JY, Chen SQ, Li HL, Duan CY (2015). Hyperuricemia is an independent predictor of contrast-induced acute kidney injury and mortality in patients undergoing percutaneous coronary intervention. Angiology.

[9] Xu X, Hu J, Song N, Chen R, Zhang T, Ding X (2017). Hyperuricemia increases the risk of acute kidney injury: a systematic review and meta-analysis. BMC Nephrol.

[10] Fang Y, Ding X, Zhong Y, Zou J, Teng J, Tang Y (2010). Acute kidney injury in a Chinese hospitalized population. Blood Purif.

[11] Hu J, Chen R, Liu S, Yu X, Zou J, Ding X (2016). Global incidence and outcomes of adult patients with acute kidney injury after cardiac surgery: a systematic review and meta-analysis. J Cardiothorac Vasc Anesth.

[12] Gami AS, Garovic VD (2004). Contrast nephropathy after coronary angiography. Mayo Clin Proc.

[13] Lepor NE (2000). Radiocontrast nephropathy: the dye is not cast. Rev Cardiovasc Med.

[14] Toprak O (2007). Risk markers for contrast-induced nephropathy. Am J Med Sci.

[15] Mueller C, Buerkle G, Buettner HJ, Petersen J, Perruchoud AP, Eriksson U (2002). Prevention of contrast media-associated nephropathy: randomized comparison of 2 hydration regimens in 1620 patients undergoing coronary angioplasty. Arch Intern Med.

[16] Wichmann JL, Katzberg RW, Litwin SE, Zwerner PL, De Cecco CN, Vogl TJ (2015). Contrast-induced nephropathy. Circulation.

[17] Kanbay M, Solak Y, Afsar B, Nistor I, Aslan G, Çağlayan OH (2017). Serum uric acid and risk for acute kidney injury following contrast. Angiology.

[18] Zuo T, Jiang L, Mao S, Liu X, Yin X, Guo L (2016). Hyperuricemia and contrast-induced acute kidney injury: a systematic review and meta-analysis. Int J Cardiol.

[19] Pelliccia F, Pasceri V, Patti G, Marazzi G, De Luca G, Tanzilli G (2018). Uric acid and contrast-induced nephropathy: an updated review and meta-regression analysis. Postepy Kardiol Interwencyjnej.

[20] Kanbay M, Segal M, Afsar B, Kang DH, Rodriguez-Iturbe B, Johnson RJ (2013). The role of uric acid in the pathogenesis of human cardiovascular disease. Heart.

[21] Kanbay M, Solak Y, Dogan E, Lanaspa MA, Covic A (2010). Uric acid in hypertension and renal disease: the chicken or the egg?. Blood Purif.

[22] Mendi MA, Afsar B, Oksuz F, Turak O, Yayla C, Ozcan F (2017). Uric acid is a useful tool to predict contrast-induced nephropathy. Angiology.

[23] Kanbay M, Jensen T, Solak Y, Le M, Roncal-Jimenez C, Rivard C (2016). Uric acid in metabolic syndrome: from an innocent bystander to a central player. Eur J Intern Med.

[24] Takir M, Kostek O, Ozkok A, Elcioglu OC, Bakan A, Erek A (2015). Lowering uric acid with allopurinol improves insulin resistance and systemic inflammation in asymptomatic hyperuricemia. J Investig Med.

[25] Abdallah E, Mosbah O, El-Shistawy S, Khalifa G, Badr M, Amin AA (2016). Evaluating the association between hyperuricemia and contrast induced-acute kidney injury in patients with acute ST-elevation myocardial infarction undergoing percutaneous coronary intervention. SM J Nephrol Therap.

[26] Elbasan Z, Şahin DY, Gür M, Kuloğlu O, Kivrak A, Içen YK (2014). Contrast-induced nephropathy in patients with ST elevation myocardial infarction treated with primary percutaneous coronary intervention. Angiology.

[27] Kurtul A, Duran M, Yarlioglues M, Murat SN, Demircelik MB, Ergun G (2014). Association between N-terminal pro-brain natriuretic peptide levels and contrast-induced nephropathy in patients undergoing percutaneous coronary intervention for acute coronary syndrome. Clin Cardiol.

[28] Li WH, Li DY, Han F, Xu TD, Zhang YB, Zhu H (2013). Impact of anemia on contrast-induced nephropathy (CIN) in patients undergoing percutaneous coronary interventions. Int Urol Nephrol.

[29] Pakfetrat M, Nikoo MH, Malekmakan L, Tabande M, Roozbeh J, Reisjalali G (2010). Risk Factors for contrast-related acute kidney injury according to risk, injury, failure, loss, and end-stage criteria in patients with coronary interventions. Iran J Kidney Dis.

[30] Toprak O, Cirit M, Esi E, Postaci N, Yesil M, Bayata S (2006). Hyperuricemia as a risk factor for contrast-induced nephropathy in patients with chronic kidney disease. Catheter Cardiovasc Interv.

[31] Madero M, Sarnak MJ, Wang X, Greene T, Beck GJ, Kusek JW (2009). Uric acid and long-term outcomes in CKD. Am J Kidney Dis.

[32] Kodama S, Saito K, Yachi Y, Asumi M, Sugawara A, Totsuka K (2009). Association between serum uric acid and development of type 2 diabetes. Diabetes Care.

[33] Sánchez-Lozada LG, Soto V, Tapia E, Avila-Casado C, Sautin YY, Nakagawa T (2008). Role of oxidative stress in the renal abnormalities induced by experimental hyperuricemia. Am J Physiol Renal Physiol.

[34] Sreenivasan J, Zhuo M, Khan MS, Li H, Fugar S, Desai P (2018). Anemia (hemoglobin ≤ 13 g/dL) as a risk factor for contrast-induced acute kidney injury following coronary angiography. Am J Cardiol.

[35] Eun Y, Han KD, Kim DH, Kim IY, Park EJ, Lee S (2019). Association between anemia and hyperuricemia: results from the Korean National Health and Nutrition Examination Survey. Sci Rep.

[36] Závada J, Hoste E, Cartin-Ceba R, Calzavacca P, Gajic O, Clermont G (2010). A comparison of three methods to estimate baseline creatinine for RIFLE classification. Nephrol Dial Transplant.

[37] McCullough PA (2008). Acute kidney injury with iodinated contrast. Crit Care Med.

[38] Gersch C, Palii SP, Kim KM, Angerhofer A, Johnson RJ, Henderson GN (2008). Inactivation of nitric oxide by uric acid. Nucleosides Nucleotides Nucleic Acids.

[39] Lisowska-Myjak B (2010). Serum and urinary biomarkers of acute kidney injury. Blood Purif.

[40] Liu Y, Tan N, Chen J, Zhou Y, Chen L, Chen S (2013). The relationship between hyperuricemia and the risk of contrast-induced acute kidney injury after percutaneous coronary intervention in patients with relatively normal serum creatinine. Clinics (Sao Paulo).

